# Quantum computing predicts particle trajectories in optical tweezers

**DOI:** 10.1038/s41377-025-01879-x

**Published:** 2025-05-22

**Authors:** Da-Wei Wang

**Affiliations:** 1 Zhejiang Key Laboratory of Micro-Nano Quantum Chips and Quantum Control, School of Physics, and State Key Laboratory for Extreme Photonics and Instrumentation, Zhejiang University, Hangzhou, China; 2 College of Optical Science and Engineering, Zhejiang University, Hangzhou, China; 3 Hefei National Laboratory, Hefei, China

**Keywords:** Quantum optics, Optical manipulation and tweezers

## Abstract

A recent study demonstrated advancements in quantum computing by applying it to address a non-Hermitian optical manipulation problem. The emergence of exceptional points and the dynamics of optically trapped single or multiple particles were simulated using a quantum computing approach.

In recent years, there has been a significant surge of interest in non-Hermitian physics^1,2^, a field that initially emerged from foundational studies in quantum mechanics. This interest has since expanded to encompass a diverse array of disciplines within the physical sciences, including classical mechanics^3^, optics^4–6^, acoustics^7^, metamaterials^8^, and topological photonics^9^. Non-Hermitian physics explores systems governed by non-Hermitian operators, which exhibit unique phenomena such as exceptional points^1,2^, PT symmetry^4,5^, and the non-Hermitian skin effect^10^. These features have opened new avenues for understanding and manipulating wave dynamics, energy transfer, and topological properties in both quantum and classical contexts, making non-Hermitian physics a vibrant and interdisciplinary area of research.

Notably, non-Hermitian physics has also been extended to the field of optical manipulation^11,12^, a discipline renowned for its versatile, contactless, and noninvasive techniques to manipulate micro particles utilizing laser beams (see Fig. 1a), particularly exemplified by the well-known optical tweezers (optical trapping) and optical binding. Given the open nature of optical manipulation, where incident laser light can impart kinetic energy to the manipulated particles, such systems inherently exhibit non-Hermitian characteristics. Both theoretical^10^ and experimental^13–15^ studies have demonstrated the presence of exceptional points (EPs, see Fig. 1b) and the unique dynamics of particles within non-Hermitian optical manipulation. Furthermore, a generalized framework termed the *non-Hermitian non-Equipartition theory* has been established^11^ to characterize the statistically averaged kinetic and “potential” energies for optically trapped particles subjected to non-Hermitian forces, extending the classical Equipartition Theorem. This theory was derived through the analytical solution of the Langevin equation and applies to the optical trapping or binding of a single particle as well as a cluster of particles, offering deeper insights into the statistical mechanics of solving such non-Hermitian systems.

**Fig. 1:**
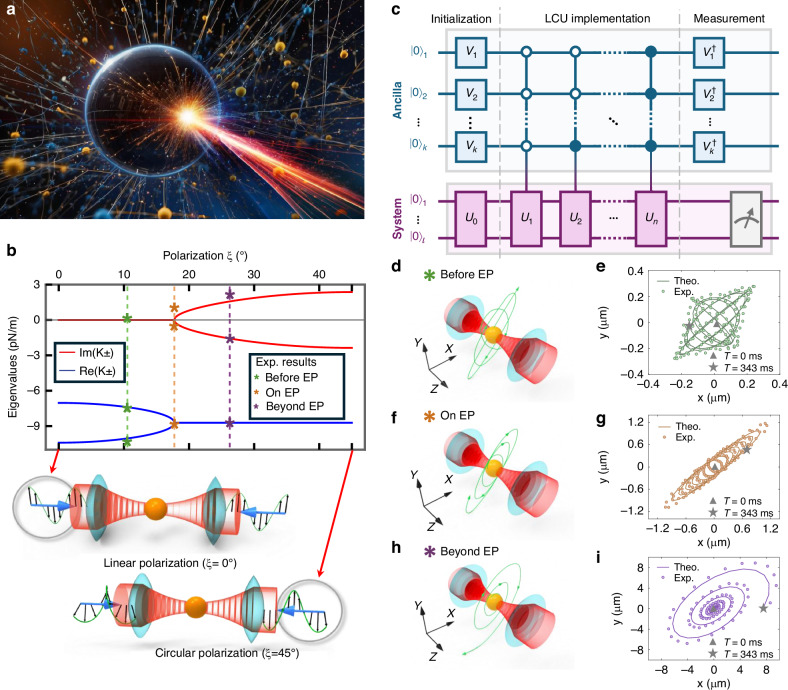
Schematics of **a** light-manipulated micro particles in a medium, (**b**) emergence of exceptional points (EPs) obtained from both theoretical predictions and quantum computing experiments, **c** general quantum circuit based on the linear combination of unitaries (LCUs), **d**, **e** theoretical and quantum-computing-experimental dynamics for an optically trapped particle before EP, **f**, **g** on EP, and (**h, i**) beyond EP

To address the challenges posed by such intrinsic non-Hermitian nature of certain systems, which complicates analytical and computational methods, quantum computing emerges as a promising alternative to predict the optically trapped particles^16^. Optical forces, inherently nonconservative due to their open-system nature, cannot be fully described by Hermitian theories. As the scale of optically bound clusters grows, analyzing and accurately simulating their dynamics becomes increasingly complex. The high controllability of quantum simulators enable them to operate directly on a minimal non-Hermitian Hamiltonian, thereby circumventing the complexities associated with traditional numerical simulations^17^. As a result, non-Hermitian dynamics have been experimentally observed in various physical systems, including ultracold atoms^18^, superconducting transmon circuits^19^, and nitrogen-vacancy centers^20^.

A recent study published in *Light: Science & Applications* by a team led by Prof. Dawei Lu and Prof. Jack Ng from the Southern University of Science and Technology introduces a novel approach to solve the non-Hermitian physics in optical manipulation with quantum computing^21^. The researchers leverage the concept of linear combination of unitaries (LCUs) to develop a quantum approach (see Fig. 1c) capable of evolving non-Hermitian Hamiltonian of qubits in a manner that accurately mimics the trajectories of optically manipulated particles within a real non-Hermitian environment. By encoding the dynamics of the trapped particle into qubits, the team was able to simulate non-Hermitian dynamics and predict particle trajectories with high accuracy. The experimental validation was conducted using a nuclear magnetic resonance (NMR) quantum processor, demonstrating the presence of non-Hermiticity and the exceptional point in a single particle system.

The study demonstrates that the quantum approach can effectively replicate the dynamics of optically trapped particles, specifically observing the transition from stability to instability at EPs (see Fig. 1b), which is evident in the displacement and velocity space of the trapped particles, underscoring the non-Hermitian nature of the system. In the single-particle experiment, stable oscillations were observed prior to the emergence of the EP (see Fig. 1d, e). Beyond this EP, the oscillations exhibited divergence (see Fig. 1h, i), indicating instability. Furthermore, the corresponding trajectory precisely on the EP was also reported (see Fig. 1f, g). The experimental results closely align with theoretical predictions, thereby validating the efficacy of the quantum approach (see Fig. 1d–i).

The method can also be extended to address the non-Hermitian physics of a many-particle optical binding scenario. They also examined the configuration of three dielectric particles that were optically bound and arranged in 1D space with their method by numerical simulation. The intensities of the two plane waves were deliberately chosen to break the reflection symmetry that produces standing wave, leading to enhanced non-Hermiticity. Upon increasing the non-Hermiticity, they observed the transition of the eigenvalues of the force matrix from real to complex and the emergence of EP, validating the quantum approach’s capability to accurately simulate non-Hermitian dynamics.

Overall, this work shows how a quantum computer can help to optimize an optical tweezer. Compared to traditional computational methods on classical computers, this work lays a foundational framework for leveraging quantum computing to explore large-scale non-Hermitian optical manipulations, offering new insights into their dynamics and control.
